# ABSL-4 Aerobiology Biosafety and Technology at the NIH/NIAID Integrated Research Facility at Fort Detrick

**DOI:** 10.3390/v6010137

**Published:** 2014-01-07

**Authors:** Matthew G. Lackemeyer, Fabian de Kok-Mercado, Jiro Wada, Laura Bollinger, Jason Kindrachuk, Victoria Wahl-Jensen, Jens H. Kuhn, Peter B. Jahrling

**Affiliations:** Integrated Research Facility at Fort Detrick, Division of Clinical Research, National Institute of Allergy and Infectious Diseases, National Institutes of Health, Fort Detrick, Frederick, MD 21702, USA; E-Mails: matthew.lackemeyer@nih.gov (M.G.L); fabiandekok@gmail.com (F.K.-M.); wadaj@niaid.nih.gov (J.W.); bollingerl@niaid.nih.gov (L.B.); kindrachuk.kenneth@nih.gov (J.K.); Victoria.Jensen@nbacc.dhs.gov (V.W.-J.); kuhnjens@niaid.nih.gov (J.H.K.)

**Keywords:** ABSL-4, aerobiology, biosafety level 4, class III biosafety cabinet, BSL-4, high-consequence viral pathogens, medical countermeasure, viral hemorrhagic fever

## Abstract

The overall threat of a viral pathogen to human populations is largely determined by the modus operandi and velocity of the pathogen that is transmitted among humans. Microorganisms that can spread by aerosol are considered a more challenging enemy than those that require direct body-to-body contact for transmission, due to the potential for infection of numerous people rather than a single individual. Additionally, disease containment is much more difficult to achieve for aerosolized viral pathogens than for pathogens that spread solely via direct person-to-person contact. Thus, aerobiology has become an increasingly necessary component for studying viral pathogens that are naturally or intentionally transmitted by aerosol. The goal of studying aerosol viral pathogens is to improve public health preparedness and medical countermeasure development. Here, we provide a brief overview of the animal biosafety level 4 Aerobiology Core at the NIH/NIAID Integrated Research Facility at Fort Detrick, Maryland, USA.

## 1. Introduction

Microorganisms infect humans through numerous routes of transmission. Viral pathogens that are dependent on direct person-to-person contact for transmission are generally contained by preventing direct physical contact between humans. Although viral pathogens that spread by direct contact can still cause disease outbreaks on a global scale (e.g., human immunodeficiency virus 1), these outbreaks usually develop in a protracted manner (decades) rather than spreading geometrically in an explosive manner. Novel pandemic threats to human populations are therefore largely relegated to microorganisms that can be transmitted by aerosols (large respiratory droplets or small particle droplet nuclei) [1]. As a result, humans infected with such pathogens may infect numerous people within larger urban settings in a very short time. Thus, complete containment of aerosolized viral pathogens may prove impossible in instances when the numbers of infected people have reached a critical limit. Viral pathogens that cause significant morbidity and lethality through their natural airborne transmission include influenza A viruses, severe acute respiratory syndrome coronavirus, human herpesvirus 3 (varicella zoster virus), and variola virus. Recently, epidemiologists and biodefense researchers are increasingly concerned that bioterrorists or other aggressors may “aerosolize” pathogens usually not transmitted through the air. In this scenario, the introduction of pathogens into the human host through an unnatural mechanism may result in modified disease courses that clinicians may fail to properly diagnose and treat. 

To address public health threats posed by natural airborne pathogens or by those artificially aerosolized, aerobiology has become a crucial component for characterization and evaluation of pathogen threats [2]. Aerobiology studies have become increasingly sophisticated over the past decade, thanks to significant technological advances made at multiple scientific institutions. Research at the NIH/NIAID Integrated Research Facility at Fort Detrick (IRF-Frederick) located in Frederick, MD, USA, focuses on high consequence emerging viral pathogens that require animal biosafety level 4 (ABSL-4) or biosafety level 4 (BSL-4) containment, including specific arenaviruses, bunyaviruses, flaviviruses, and filoviruses. Work with these NIAID Priority Pathogens and/or CDC Bioterrorism Agents requires rigorous and specialized staff training, strict biosecurity/biosurety oversight, and the highest level of biocontainment [3,4,5]. Biosafety precautions for ABSL-4/BSL-4 experiments are outlined in the Biosafety in Microbiological and Biomedical Laboratories (BMBL) manual and several other publications [6,7,8]. Such precautions are implemented through facility-specific standard operating procedures. The primary focus of the IRF-Frederick is to facilitate the development and/or evaluation of medical countermeasures against high consequence viral pathogens and ease the transition of candidate therapeutics or vaccines to clinical licensure. To accomplish this task, the IRF-Frederick has been constructed to mimic an intensive care unit for animals experimentally infected with high consequence viral pathogens [9]. Here we describe the Aerobiology Core of the IRF-Frederick and the safety requirements and procedures that govern aerobiology.

## 2. Aerobiology Biosafety and Technology

### 2.1. Class III Biological Safety Cabinet

**Figure 1:**
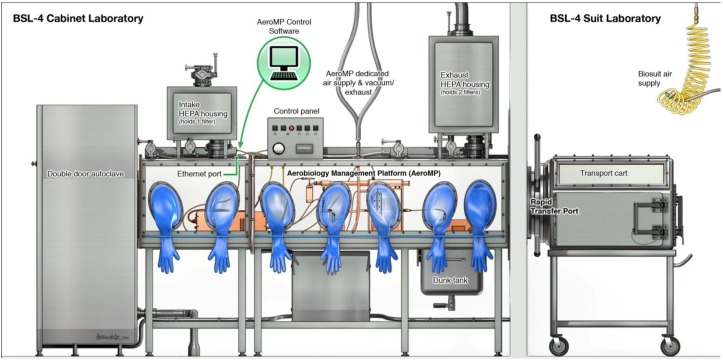
Schematic presentation of the Class III Biosafety Cabinet setup at the IRF-Frederick. The Class III biosafety cabinet (BSC) is a hermetically sealed stainless steel cabinet containing an ABSL-4 environment under negative pressure (currently in static state for easy viewing) within an ABSL-4 cabinet laboratory. Materials can be introduced into the BSC by staff working in the ABSL-4 cabinet laboratory through an under-cabinet-mounted stainless steel tank containing the disinfectant Micro-Chem Plus^®^ (commonly referred to as a “dunk tank” in ABSL-4 or BSL-4 settings). Because the BSC is built into the wall separating the cabinet laboratory from an ABSL-4 suit laboratory, materials, animals, and viral pathogens can also be moved into the BSC from the ABSL-4 suit laboratory side using a transport cart and a Rapid Transfer Port (RTP). The contents within the BSC can be manipulated from the outside by researchers wearing various types of synthetic rubber gloves, specifically neoprene/chlorosulphonated polyethylene. Contents, excluding infectious samples, are removed from the BSC after sterilization through a double-door autoclave or disinfection via the dunk tank.

**Figure 2:**
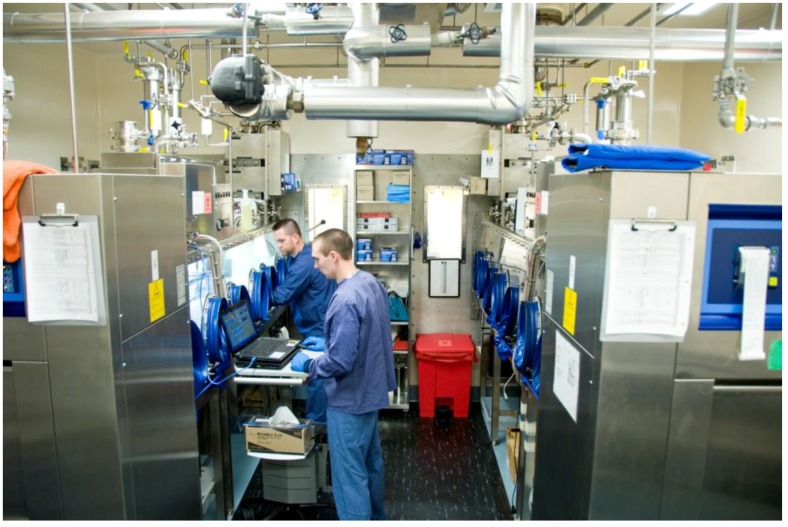
The IRF-Frederick Aerobiology Core Class III BSC laboratory. Shown are the IRF-Frederick Aerobiology Core’s two Class III BSCs within the ABSL-4 cabinet laboratory. One researcher is manipulating the contents of one of the Class III BSCs via synthetic rubber gloves. The other researcher is monitoring the parameters of the experiment electronically.

**Figure 3:**
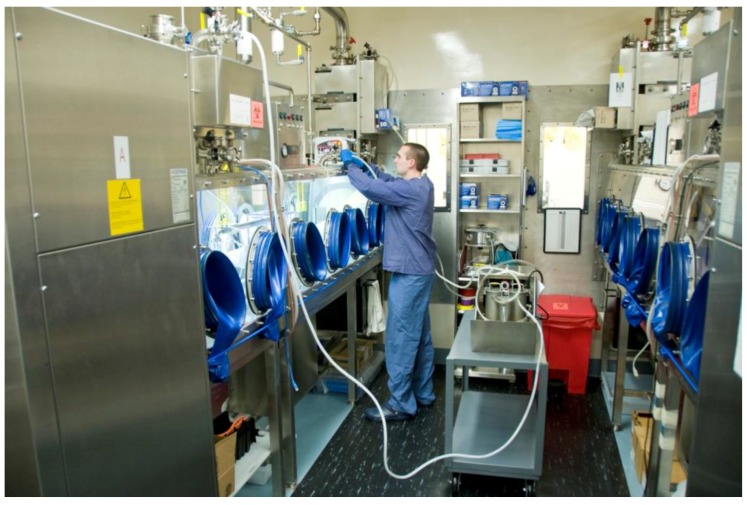
Class III BSC paraformaldehyde gas decontamination setup. After an experiment, a researcher connects a decontamination machine to the BSC through designated external ports on top of the glove box. From a paraformaldehyde gas generator (Certek, Durham, NC, USA), the gas is pumped into the BSC and decontaminates the BSC after an appropriate contact time (*i.e.*, 6 h). The gas is then neutralized by ammonium bicarbonate for an additional 6 h (12 h total decontamination). Biological indicators are placed throughout the BSC and the results are recorded and analyzed by staff.

Aerosol studies at the IRF-Frederick are performed within two negative-pressure (minimum: 125 Pa [−0.5 inches water gauge]; operational: 250 Pa [−0.75 to −1.0 inches water gauge]), high grade stainless steel Class III biological safety cabinets (BSCs) situated within an ABSL-4 cabinet laboratory (Figure 1 and Figure 2) [10,11]. The Class III BSC provides a primary barrier of protection and a safe, controlled, ABSL-4 or BSL-4 environment in which researchers perform viral pathogen characterization or *in vivo* experiments. Decontamination of the cabinets must take place between experiments to facilitate safe maintenance and/or repairs and to prevent cross-contamination between subsequent studies. The method of choice for decontamination at the IRF-Frederick is paraformaldehyde gas, as the gas is effective and easy to use in Class III BSCs [12]. All decontamination procedures follow standard operating procedures. Successful decontamination of a Class III BSC is documented from the results of biological indicators that validate agent inactivation (Figure 3). 

**Figure 4:**
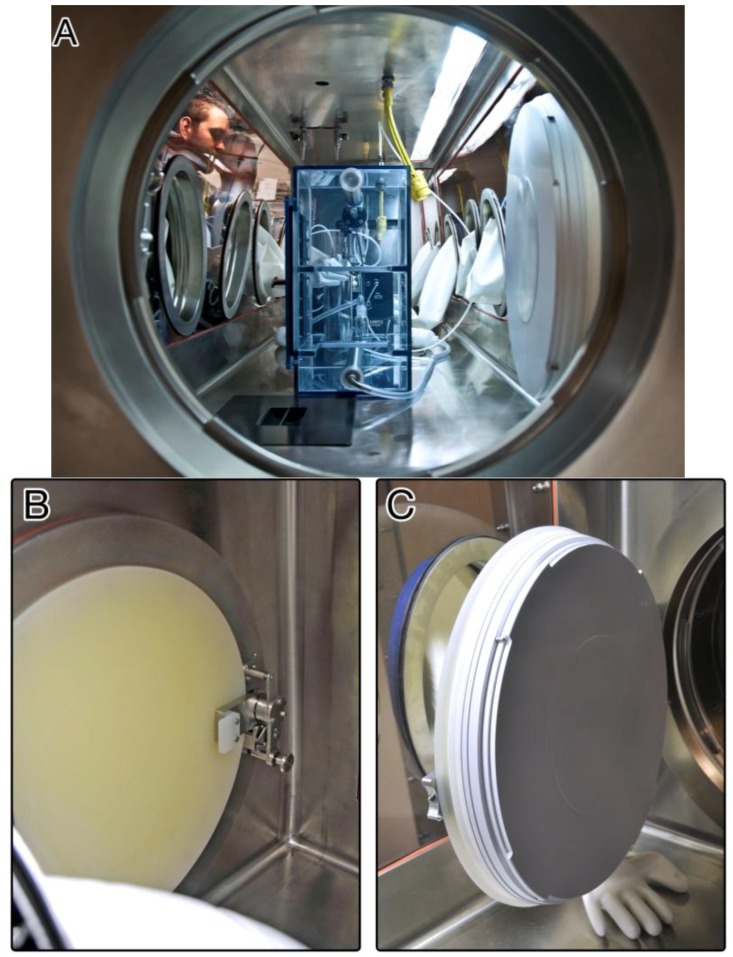
The IRF-Frederick Class III BSC laboratory Rapid Transfer Port. (**A**) View into one of the IRF-Frederick Aerobiology Core Class III BSCs through the Rapid Transfer Port (RTP) connecting the ABSL-4 environment within the BSC to the ABSL-4 suit laboratory. Shown on the left are two researchers in the ABSL-4 cabinet laboratory manipulating the Automated Aerosol Management Platform (AAMP) connected to a whole-body aerosol exposure chamber in the Class III BSC using synthetic rubber gloves; (**B**) View of the RTP interlocking handle, port door closed; (**C**) View of RTP door seal, port door open.

**Figure 5:**
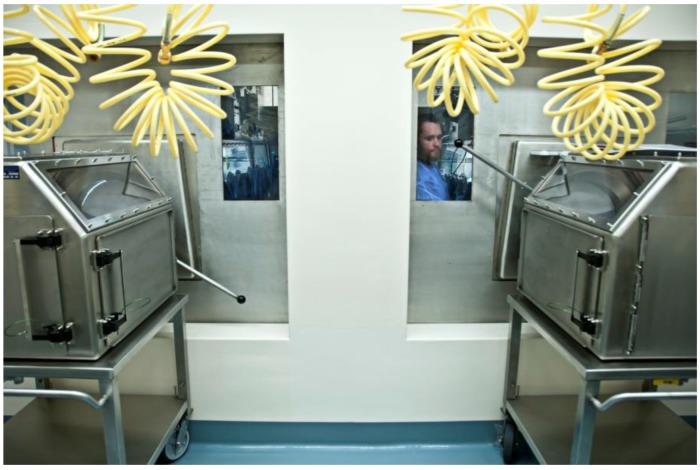
ABSL-4 suit laboratory transport carts. Two specialized transport carts within the ABSL-4 suit laboratory are docked to the Class III BSC at the Rapid Transfer Port. Through windows in the wall between the ABSL-4 suit laboratory and the Class III BSC, researchers see into the adjacent ABSL-4 cabinet laboratory containing the Class III BSCs and communicate with staff.

**Figure 6:**
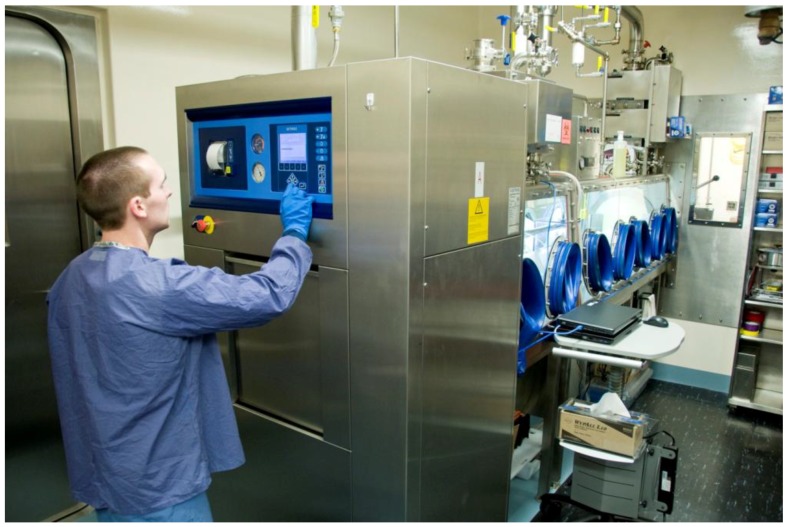
Interlocking double-door autoclave attached to the Class III BSC. Used contents and waste generated during an experiment inside the BSC must be sterilized after use. A researcher is selecting a pre-programmed autoclave cycle to ensure the contents within the autoclave chamber are noninfectious when the outer door is eventually opened. The door located nearest to the researcher cannot be opened until a full sterilization cycle has been completed. Biological indicators inside the autoclave chamber will be analyzed to determine agent inactivation after the sterilization process.

A Rapid Transfer Port (RTP) connects the Class III BSC to the adjacent BSL-4 suit laboratory (Figure 4) [13]. Specialized transport carts are used to pass experimental animals and materials within the ABSL-4 suit laboratory through to the Class III BSC (Figure 5). The BMBL manual mandates that all Class III cabinet laboratories have an attached autoclave and equivalent decontamination method, such as a pass-through tank filled with disinfectant (dunk tank) [6]. At the IRF-Frederick, materials are sterilized and passed in and out of the cabinet through a double-door autoclave (Figure 6). An interlocking door mechanism ensures that only one autoclave door can be opened during Class III BSC use. This mechanism also prevents the outer door of the autoclave from being operational until a decontamination cycle has taken place. Waste samples are sterilized and disposed of after an autoclave cycle consisting of the appropriate temperature, pressure, and contact time (121.1 °C, 205.7 kPa, 2 h).

**Figure 7:**
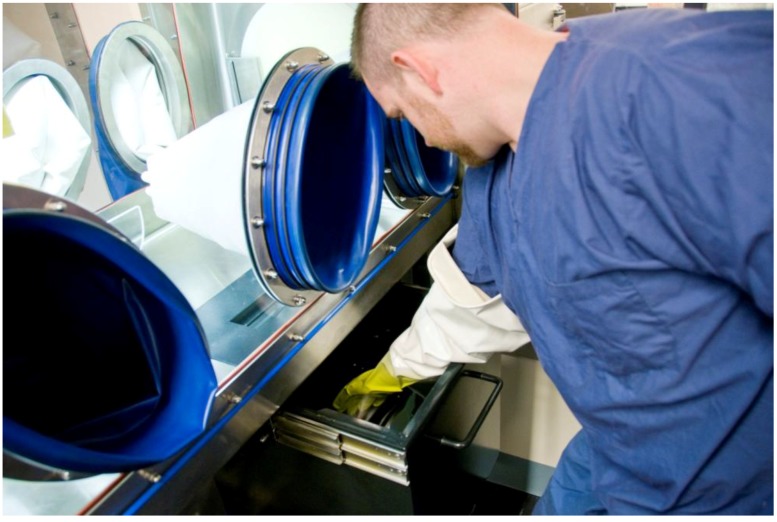
Dunk tank filled with disinfectant attached to the Class III BSC. A liquid barrier created by the appropriate disinfectant protects the researcher by ensuring that contaminants within the BSC are fully contained. Supplies are passed through the dunk tank and into the BSC. A black lid covers the base of the BSC and prevents liquid from splashing into the experimental area.

A chemical immersion dunk tank, which is filled with the appropriate level and concentration of disinfectant (≈3,500 μS/cm of Micro-Chem Plus^®^), is attached below the Class III BSC. Disinfectant in the tank is a liquid barrier of protection between the inside of the cabinet and the outside operator space (Figure 7). Dunk tanks provide the researcher with easy access to supplies during experimentation when the Class III BSC is in use. Within an ABSL-4 cabinet laboratory, non-infectious items/tools may be passed into the contaminated Class III BSC but must remain inside until full gas decontamination has taken place or until the items/tools are disinfected in the dunk tank mounted under the floor of the Class III BSC. Appropriate contact time of items/tools with the disinfectant in the tank is 10 min. If samples are transported out of the Class III BSC for any reason, then appropriate barriers and disinfectant should be used to provide protection and containment. Experimental samples kept for further analysis and study must be passed from the Class III BSC back into the BSL-4 suite through the RTP into a transport cart. 

Lastly, a dedicated High-Efficiency Particulate Air (HEPA) filtration system is used in conjunction with the general laboratory exhaust system, known as the “HEPA deck.” Air entering the Class III BSCs is filtered once, whereas outgoing air is filtered twice before being discharged to the facility’s HEPA deck exhaust system [6,9]. The two exhaust HEPA filters on the Class III BSCs are installed “in series” and are certified independently by use of the disperse oil particle scan method. After gas decontamination of HEPA filters, all HEPA filters are tested, and a negative result indicates that the filters are “clean” or “non-infectious”. HEPA filters are also tested for leaks and certified annually to ensure correct functionality [10].

### 2.2. Personnel and Protective Equipment

Through a series of 24.5 cm (10 inch) glove ports on the sides of the Class III BSC, the researcher handles BSL-4 materials within the cabinet (Figure 1, Figure 2, and Figure 4). The ports are furnished with extended, arm-length synthetic rubber gloves that range in thickness and material depending on scientific application and disinfectants used. In addition to the integrated gloves, all personnel must wear a single layer of latex/nitrile gloves, autoclavable or disposable long sleeve scrubs, and appropriate footwear when working in the Class III BSC. The latex/nitrile gloves are a protective barrier between the user and the Class III BSC in the event there is a breach of containment within the integrated synthetic rubber gloves. Upon completion of use, the latex/nitrile gloves are disinfected with 5% Micro-Chem Plus^®^ before disposal into double biohazardous trash bags in a step can. When exiting the laboratory, staff must take a shower, and all used scrubs must remain inside the containment portion of the change room. The scrubs are placed into laundry bags, autoclaved outside of the laboratory, and then laundered. Additionally, all footwear remain inside the containment portion of the changing room. 

### 2.3. Automated Aerosol Management Platform

**Figure 8:**
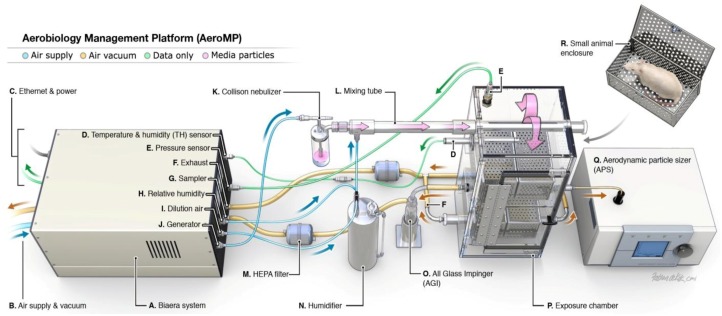
Schematic presentation of the Automated Aerosol Management Platform. The AAMP is fully contained within the Class III BSC. Compressed air under negative pressure enters the aerosol unit, which is controlled by mass flow controllers and experimental parameters dictated by the researcher on a laptop (not shown). Droplets produced by aerosol generation pass through a mixing tube, are diluted with additional air, and dried. This conditioning of particles ensures that the particles are monodispersed prior to entry into the whole body chamber. Simultaneously, the vacuum pulls air through the whole body chamber ensuring that newly generated particles pass through the chamber while maintaining a dynamic system. A biosampler or cascade impactor and particle sizer are attached to the whole body chamber for collection and analysis. The overall flow of the system is shown by the directional arrows.

The IRF-Frederick Aerobiology Core employs an Automated Aerosol Management Platform (AAMP) AeroMP^®^ (Biaera Technologies, Hagerstown, MD, USA) to control and monitor aerosol experimentation (Figure 8). This platform was developed at the United States Army Medical Research Institute of Infectious Diseases (USAMRIID) maximum containment laboratory [14]. All AAMP components are selected and manufactured to withstand repeated decontamination with paraformaldehyde gas and hydrogen peroxide vapors. The system does not require tools to assemble and is designed without any cut, catch, or tear points to avoid compromising the integrity of protective gloves worn by staff. Multiple institutions have successfully used this technology, and the results are well documented [14,15,16,17,18]. 

The AAMP components are integrated with the Class III BSC (Figure 4A). This turnkey unit electronically controls and monitors numerous parameters that can influence an aerosol delivery, such as temperature, relative humidity, aerosol particle size, particle concentration, exposure duration, exposure chamber pressure, input airflow, and exhaust airflow in real time [19]*.* Researchers set parameters that affect aerosol delivery to optimum settings that will maintain the stability and viability of infectious virus particles over the course of the experiment. Communication between the control software, which runs on a laptop in the ABSL-4 cabinet laboratory, and the AAMP is accomplished with Category 5 Ethernet cables (CAT 5), USB connections, or Bluetooth technology. The Class III BSC is equipped with an integrated, connective port between the AAMP and the user’s laptop to maintain the integrity of biocontainment. 

The AAMP attaches to dedicated air supply and vacuum ports, which are hard plumbed to the Class III BSC. Thus, additional penetrations for equipment into the Class III BSC that potentially compromise containment are not needed. The exhaust ports of the AAMP exposure chamber also contain in-line HEPA filters, ensuring that all expelled particulates are trapped and contained. 

The AAMP consists of aerosol generator(s), exposure chamber(s), aerosol biosampler(s), and aerodynamic particle sizer(s). A variety of aerosol generators can be used in conjunction with the AAMP in the Class III BSC. The IRF-Frederick generally uses a Collison nebulizer (BGI Inc. Waltham, MA, USA) to generate small aerosol particles from liquid media containing virus. To generate large particles, the Centered flow Tangential Aerosol Generator (CenTAG) (CH Technologies, Westwood, NJ, USA) is used and controlled by a syringe pump and AAMP software. Once the particles are expelled from the aerosol generators, they are diluted with additional air, dried to an optimal size within the mixing tube, and subsequently delivered to the exposure chamber.

An exposure chamber is connected to the AAMP unit. The type of exposure chamber used is dependent on the test subject (type of animal) and desired aerosol exposure (head only, nose only, or whole body). Pressure within the exposure chamber can be positive or negative, depending on parameters set by the researcher. From the perspective of safety, negative pressure is preferred to ensure all generated particulates are drawn towards the exposure chamber and through the exhaust ports. 

Aerosol particles are analyzed during the exposure for particle size distribution by an aerodynamic particle sizer or cascade impactor and for virus aerosol concentration by a biosampler. The aerodynamic particle sizer provides high-resolution, real-time aerodynamic measurements of particles containing a mass median aerodynamic diameter ranging from 0.5 to 20 µm. A few readings from the particle sizer gives a snapshot of the total particle count in the sampled air, mass median aerodynamic diameter of the particles, and geometric standard deviation. The cascade impactor (e.g., Cascade Impactor, In-Tox Products) analyzes particle size continuously during the exposure. The researcher analyzes particle size (mass) within a dedicated size range. As the particle size decreases, the ability to travel further through the device increases. The researcher determines the size range of the majority of generated particles from mass accumulation of particles collected onto each stage (collection plate with culture medium) of the cascade impactor (e.g., seven stages for the Cascade impactor). From the ability to generate particles of different sizes and to evaluate the particle size distribution in relation to disease course and pathogenesis, the researcher can estimate the deposition of particles within the respiratory tract. The deposition pattern is dependent on particle size, density, shape, airway geometry, and breathing pattern, as well as the mechanisms of deposition (impaction, settling, diffusion, interception, and electrostatic deposition) [20]. In general, small particles are preferred for penetration to the tracheobronchial and pulmonary regions of the lung, with emphasis on the lower respiratory tract [21]. Larger particles will distribute close to the head airway region via impaction [20]. 

Virus aerosol concentrations are continuously sampled by a biosampler (e.g., All Glass Impinger, SKC Biosampler) during an exposure. The particles collected by the biosampler are grown in culture medium to determine the titer of the aerosolized viral pathogen via plaque assay. Alternatively, RNA or DNA is extracted, and genome copies are quantified via polymerase chain reaction. Samples generated from aerosol exposure must be evaluated with inside a Class II BSC within the ABSL-4 suit laboratory as they are considered to contain infectious viral pathogens.

### 2.4. Experimental Preparation

Preparation for aerosol experiments generally begins 2 to 7 days prior to study initiation. All necessary pieces of equipment used for the aerosol exposure and safety devices housing the equipment are calibrated for safe use, certified for functionality once each year, and parameters checked for inconsistencies prior to commencing each experiment [22]. This equipment includes, but is not limited to, aerosol generators, biosampling devices (e.g., flow rate), particle sizers, exposure chambers (e.g., leak detection), flow controllers, air and vacuum supply, autoclaves, and the Class III BSC itself. The AAMP automatically monitors and maintains environmental conditions (e.g., temperature, relative humidity, negative pressure) within the exposure chamber, balances the system airflows (e.g., biosampler airflow, airflow into and out of exposure chamber, flow rate for aerodynamic particle sizer), and controls biosampling and particle sizing. These aerobiology parameters are programmed into the AAMP before initiation of the experiment.

Prior to animal experimentation, aerosol generation, biosampling, particle size distribution, and plethysmography protocols are validated and optimized. To optimize biosampling parameters and maximize recovery, aerosolized virus is introduced into the exposure chamber to quantify loss of virus from the rigors of particle generation (e.g., mechanical disruption during aerosol generation, generation of droplet size) and sample collection. In addition, loss of virus may also be the result of other experimental conditions (e.g., loss of viability through dehydration, mutant instability, temperature, relative humidity, exposure time duration). Optimizing parameter settings (aerosol characterization) will ensure viable virus aerosol concentrations during an actual experiment and experimental reproducibility [22,23]. During a calibration or sham experiment, the initial virus aerosol concentration is increased logarithmically, and the actual virus aerosol concentration delivered is measured. The ratio, or spray factor, of the delivered virus aerosol concentration to the initial virus aerosol concentration generated expresses the viral loss for a particular agent. 

For animal experiments, the initial aerosol concentration of virus is estimated based on the viable aerosol concentration (from biosampler or cascade impactor) and the spray factor. Particle size distribution and mass are analyzed depending on scientific goals and results. Aerosol equipment parameters are tweaked to obtain a monodisperse particle size and mass.

Plethysmography equipment is calibrated for baseline parameters and checked for structural integrity and functionality. Minute volume is calculated from a calibration curve that is produced by injection of a known volume of air into the plethysmograph over a wide range of simulated respiratory frequencies. Such calibrations can capture simulated animal-to-animal variations in pulmonary function but cannot account for changes in respiratory function caused by the use of anesthetics, differing individual animal metabolisms, respiratory effects caused by the agent under investigation, or changing stresses on the animal during exposure [14]. 

### 2.5. Experimental Procedures

During an actual experiment, animals undergo plethysmography immediately prior to aerosol exposure within the Aerobiology ABSL-4 suit laboratory that abuts to the Class III BSC (Figure 1). Animals are placed into a transport cart by staff wearing a positive-pressure suit. Then animals are passed through a rapid transfer port and are placed into the exposure chamber of the AAMP. 

The inhaled dose for the set duration of the exposure can be back-calculated using either predetermined, formula-generated minute volume values or actual minute volume values obtained via plethysmography. In experiments involving smaller animals, the inhaled dose is calculated using body-weight-based formulas to estimate respiratory minute volume. With one such formula, Guyton’s formula, the respiratory minute volume is estimated by cc/min = 2.1 × (body weight in grams)^0.75^ [24]. In larger animals, the inhaled dose is calculated using a simplified formula D = R × C_aero_ × T_exp_. D is the presented or inhaled dose (e.g., virus plaque-forming units), R is the respiratory minute volume (L/min) measured by plethysmography, C_aero_ is the aerosol concentration (plaque forming units/L), and T_exp_ is the duration of the exposure (min) [14,23]. In the future, the respiratory minute volume will be monitored continuously. Then, exposure time will be varied so that the same inhaled dose could be delivered to each animal, as the minute volume of larger animals can vary widely [14]. By monitoring the respiratory function during the experiment, the researcher can control the amount and duration of the inhalation procedure, rather than using predetermined estimates of time of exposure. The AAMP will then terminate the exposure when the target dose of the virus is achieved. 

Experiments involving animals are more complex than calibration experiments and must be coordinated with the veterinary technical staff to provide anesthesia and monitoring of animal vital signs. The AAMP notifies the researcher when any environmental parameter (e.g., temperature, relative humidity, pressure, aerosol concentration, flow velocity) falls outside of upper and lower limits defined by the researcher [14,25].

## 3. Conclusion

Aerosol studies are a necessary part of research on infectious agents that are either naturally or intentionally spread by the respiratory route. Appreciable strides have been made within the field of aerobiology, particularly concerning safety and reproducibility of experimental parameters within maximum biocontainment. Through the integration of a Class III BSC and the AAMP within high containment, an efficient, reliable, and safe environment is ensured for *in vivo* research with high consequence viral pathogens. The Aerobiology Core at the IRF-Frederick seamlessly integrates with other state-of-the-art capabilities, such as ABSL-4 medical imaging [9], to facilitate the establishment or refinement of animal models and the evaluation of candidate medical countermeasures.
